# Development of reverse-transcriptase, real-time PCR assays to distinguish the Southern African Territories (SAT) serotypes 1 and 3 and topotype VII of SAT2 of Foot-and-Mouth Disease Virus

**DOI:** 10.3389/fvets.2022.977761

**Published:** 2022-09-20

**Authors:** Taeyo Chestley, Patrycja Sroga, Michelle Nebroski, Kate Hole, Hussaini Ularamu, Oliver Lung, Charles Nfon

**Affiliations:** ^1^National Centre for Foreign Animal Disease, Canadian Food Inspection Agency, Winnipeg, MB, Canada; ^2^National Veterinary Research Institute, Vom, Plateau State, Nigeria

**Keywords:** Foot-and-Mouth Disease Virus, FMDV, Southern African Territories, serotyping, detection, real-time reverse transcriptase polymerase chain reaction

## Abstract

Foot-and-Mouth Disease Virus (FMDV), the causative agent of Foot-and-Mouth Disease, is a highly feared, economically devastating transboundary pathogen. This is due to the virus' extremely contagious nature and its ability to utilize multiple transmission routes. As such, rapid and accurate diagnostic testing is imperative to the control of FMD. Identification of the FMDV serotype is necessary as it provides the foundation for appropriate vaccine selection and aids in outbreak source tracing. With the vast genetic diversity, there is a desperate need to be able to characterize FMDV without relying on prior knowledge of viral serotypes. In this study, the Neptune bioinformatics tool was used to identify genetic signatures specific to each Southern African Territories (SAT) 1, 2 and 3 genomes but exclusionary to the other circulating FMDV serotypes (A, O, Asia1, and the heterologous SAT1, SAT2 and/or SAT3). Identification of these unique genomic regions allowed the design of TaqMan-based real-time reverse transcriptase PCR (rRT-PCR) primer/probe sets for SAT1, SAT2 and SAT3 viruses. These assays were optimized using prototypic FMDV cell culture isolates using the same reagents and thermocycling conditions as the FMDV pan-serotype 3D rRT-PCR assay. Cross-reactivity was evaluated in tandem with the FMDV pan-serotype 3D rRT-PCR utilizing representative strains from FMDV serotypes A, O, Asia1, SAT1, SAT2 and SAT3. The SAT1, SAT2, and SAT3 primer/probe sets were specific for the homologous serotype and exclusionary to all others. SAT1 and SAT3 primer/probe sets were able to detect several topotypes, whereas the SAT2 assay was revealed to be specific for topotype VII. The SAT2 topotype VII specificity was possibly due to the use of sequence data deposited post-2011to design the rRT-PCR primers and probes. Each assay was tested against a panel of 99 bovine tissue samples from Nigeria, where SAT2 topotype VII viruses were correctly identified and no cross-reactivity was exhibited by the SAT1 and 3 assays. These novel SAT1, SAT3 and SAT2 topotype VII rRT-PCR assays have the potential to detect and differentiate circulating FMD SAT viruses.

## Introduction

Foot-and-Mouth Disease (FMD) is a highly contagious viral disease affecting even-toed ungulates. While mortality rates are often low in adult animals (1%−5%) they are inversely correlated with age and have been reported to be up to 94% in lambs, 80% in calves, and 100% in suckling piglets (1). However, the disease is devastating to the animals as they lose their ability to eat, drink, and walk due to extremely painful lesions. These debilitating effects subsequently lead to many direct losses including, lower weight gains, decreased milk production and a loss in draught power (2).

The characteristic clinical manifestation of FMD is the formation of vesicles in the mouth and on the feet of afflicted animals, often accompanied by fever and profuse salivation. Disease signs appear between 1 and 14 days after initial infection depending on infectious dose, transmission route and housing (3). Suspicion of FMDV infection must be confirmed through laboratory diagnosis as signs are nebulous and clinically indistinguishable from other vesicular diseases.

The causative agent of FMD is the Foot-and-Mouth Disease Virus (FMDV), a member of the *Picornaviridae* family in the *Apthovirus* genus. Virions are non-enveloped and utilize capsid proteins to encase a ~8.3 kilobase single-stranded, positive-sense RNA genome (4). Extensive genetic heterogeneity is a key characteristic of FMDV and is reflected at both the genetic and antigenic levels. Seven immunologically distinct serotypes exist and include A, O, C, Asia1, Southern African Territories (SAT)1, SAT2 and SAT3, however, serotype C has not been detected since 2004 (5). Within these serotypes, there are many subtypes/topotypes and lineages. There is no antigenic cross-reactivity between serotypes, and this is often also extended to subtypes (6).

Preparedness through having established rapid, sensitive, and readily-available diagnostic tests is critical to FMDV control. Accurate tests and quick turnaround times are imperative to cease the spread and manage unnecessary animal culling. Most FMDV diagnostic testing methods that detect viral antigen or genomic RNA are serotype independent and verify FMDV presence. Pan-serotype real-time reverse transcriptase PCR (rRT-PCR) that detects either the 3D or internal ribosome entry site (IRES) portion of the FMDV genome are highly sensitive and accurate first-line diagnostic tests (7–9). These tests are capable of determining the presence of the FMDV genome only, therefore, in order to fully characterize an FMDV incursion, it is essential to identify the virus serotype.

FMDV serotyping provides the necessary first step in establishing a VP1 Sanger-based sequencing approach and identifying an appropriate FMDV vaccine. The FMDV antigen detection ELISA (Ag-ELISA) is the most common methodology for identifying FMDV serotype. The Ag-ELISA consists of seven serotype-specific polyclonal antibodies that capture the FMDV capsid antigen which is then detected *via* a serotype-specific guinea pig antibody. A major pitfall of the Ag-ELISA is the low sensitivity of 80%−90% for positive bovine samples and <80% for porcine samples (10). Sensitivity issues also extend to the sample source. While vesicular fluid and vesicular epithelium are preferred samples utilized in the Ag-ELISA as viral titers are the highest, less-invasive samples such as blood, oropharyngeal fluids, and mucosal swabs may lead to false negatives or require additional passage in cultured cells (3, 11).

Currently, sequencing of the 1D (VP1 protein) region of the FMDV genome is typically accomplished utilizing Sanger termination sequencing methodology. The VP1 capsid protein contains a surface exposed G–H loop formed by residues 140–160 of the βG and βH chains and this exposure results in its constant evolution (12, 13). This lack of genetic conservation provides enough sequence information to differentiate FMDV to the strain level. However, current FMDV Sanger sequencing protocols require prior knowledge of the serotype/subtype sequence, and the lengthy protocol requires two amplification procedures as well as costly reagents and equipment (14). Next-generation sequencing (NGS) methodologies are powerful tools to generate sequence data but some technologies, such as the widely used Illumina short-read sequencing, are time-consuming and require expensive equipment. Generally, NGS requires more specialized technical and bioinformatics expertise and can have longer turnaround times when compared with PCR-based methods.

Real-time conventional PCR methods, in combination with size differentiation based on agarose gel electrophoresis, were the first attempts to utilize PCR technology to identify FMDV serotypes (15–24). However, several of these assays demonstrated serotype cross-reactivity (15, 16). Issues with strain sensitivity were also noted as several strains within a serotype eluded detection (19, 21). In order to produce assays with higher sensitivity and specificity, the approach to target specific geographic regions and thus specific FMDV pools was adopted. As an example, Giridharan et al. described a method where primer sets were designed based on isolates circulating in India that successfully detected O, A, Asia, and C serotypes (22). In another example, using TaqMan-based rRT-PCR, Reid et al. (25) designed FMDV serotyping assays directed to Middle Eastern O, A, and Asia1 viruses. Jamal and Belsham in 2015 (26) designed primer/probe sets capable of distinguishing FMDV serotypes A, O, and Asia1 circulating in pools present in West Eurasia. Likewise, Bachanek-Bankowska et al. were able to discern FMDV A, O, SAT 1 and 2, restricted to viruses found in East Africa (27). El Bagoury et al. (28) produced rRT-PCR assays capable of detecting and distinguishing O and SAT3 viruses circulating in Egypt. Several other lineage-specific FMDV rRT-PCR assays have been reported (25, 29–32).

With the constant emergence of new FMDV strains and variants contributing to the already vast genetic diversity, there is a need to consistently develop assays capable of identifying FMDV serotypes. In this study, an innovative bioinformatics tool, Neptune, was used to generate genetic signatures that were specific to the FMDV SAT1, SAT2, and SAT3 serotypes. Degenerate Taq-Man-based rRT-PCR assays were designed to both detect and differentiate FMDV SAT1, SAT3, and topotype VII of SAT2. Serotyping assays were optimized to utilize the same reagents and thermocycling conditions as the previously validated pan-serotype 3D rRT-PCR assay.

## Materials and methods

### FMDV samples

#### Cell culture isolates

Viruses utilized in this study were obtained from the World Reference Laboratory for FMDV, The Pirbright Institute, UK, and from the National Veterinary Research Institute (NVRI), Vom, Nigeria. FMDV isolates were propagated in either baby hamster kidney 21 (BHK-21), fetal porcine kidney (LFBK-α_v_β_6_), porcine kidney (IB-RS-2) or primary lamb kidney (LK) cell lines as previously described (33–35). Viral isolates were stored at −70°C until use.

#### Clinical field samples

Tissue samples from FMDV – infected cattle in Nigeria were collected by NVRI veterinarians, stored at −70°C, and eventually transported to NCFAD with the cold chain maintained. A 10% tissue suspension was prepared in sterile phosphate-buffered saline (PBS) using the Precellys Lysing Kit (BER-P000918LYSK0A0, ESBE Scientific) and the Precellys 24 dual tissue homogenizer as previously described (36).

### Identification of FMDV SAT serotype-specific genetic signatures

Specific FMDV SAT1, SAT2 and SAT3 genetic signatures capable of serotype identification and differentiation were identified by the Neptune bioinformatics tool, version 1.2.5 (37). Neptune analysis was performed utilizing six comparisons representing six of the seven FMDV serotypes [serotype C was excluded as it is extinct (5)]. The input required for the Neptune tool is that two arguments are presented. The first is a list of nucleotide acid sequences that are the inclusion group and the second is a list of sequences defined as the exclusion group. FMDV sequences representing the homologous target serotype populated the inclusion group and sequences from the remaining six heterologous serotypes populated the exclusion group. For SAT1, the inclusion group consisted of 510 sequences and the exclusion group contained 6,986 sequences, SAT2 758 vs. 6,738 and SAT3 115 vs. 7,381. The generated file of interest is a FASTA file called “consolidated.fasta.” Each line of the output file contains an identified genetic signature accompanied by the overall Neptune score, the values for which are based on the inclusion and exclusion group scores that are used to calculate the overall score. Also included is the accession number for the reference sequence that the marker is based on and the position in that reference sequence that the marker begins at. Neptune scores measured genetic signature confidence based on a positive value that represents the inclusion group component and a negative value representing the exclusion group component. Scores were then used to rank the produced genetic signatures by sensitivity and specificity. The signature sequences produced by Neptune for FMDV SAT1, SAT2 and SAT3 were all located in the highly variable VP1 region of the genome and are listed in Table 1.

**Table 1:** Top ranking FMDV SAT1, SAT2 and SAT3 serotype-specific genetic signatures generated by Neptune version 1.2.5 software.

**FMDV serotype**	**Gene**	**Genome location**	**Sequence (5^′^ → 3^′^)**
SAT1	1D (VP1)	3200	CTGAACCAGTCACAACTGATGCCTCACAACATGGTGGTAACGCCCGTCCCACACGGCGATACCACACCAATGTTGAGTTCTTGCTTGACCGTTTCACGCTCATAGGCAAGACACACAACAACAAAATGGTTTTGGACATGCTACGGACCGAGA
SAT2	1D (VP1)	3600	CCGATGTCGTCACGACCGGCCCTGCCACACACGGTGGTGTTGCAAACACTGCGCGACGTGCCCACACAGACGTCGCTTTCTTGCTGGATCGCAGCACACACGTGTACACCAACAAAACGTCATTCAGCGTCGATCTCATGGAAACAAAGG
SAT3	1D (VP1)	3549	CAACGGATCCTGTAAATACACCAAAACGCGAAGTGTTGGCCCGCGCCGTGGAGACTTGGCNACGCTGGCACAACGCGTAGAAACTGAGCAAGCAAGGTGTATACCCACGACATTCAACTTCGGTCGTTTGTTGTGTGATTCAGGTGAGGTGTACTACCGCATGAAGCGA

Genomic numbering corresponds to the consensus sequences generated from MAFFT alignments of 286 FMDV SAT1 sequences, 378 SAT2 sequences and 50 SAT3 sequences.

#### FMDV SAT1, SAT2, and SAT3 serotype-specific primer/probe design

Primers and probes designed to identify and differentiate between FMDV serotypes SAT1, SAT2 and SAT3 were based on the signature sequences with the highest score produced by the Neptune bioinformatics tool. To facilitate serotype inclusive primer/probe design, FMDV VP1 and full-length genome sequences belonging to the SAT1, SAT2, and SAT3 serotypes were retrieved from the National Center for Biotechnology Information (https://www.ncbi.nlm.nih.gov/). Sequences collected were limited to those deposited between 2011 and September 2021. This included 286 SAT1 sequences, 378 SAT2 sequences and 50 SAT3 sequences. Multiple alignments were performed using Geneious software, version 11.1.5, and the MAFFT version 7.450 algorithm (38–40). FMDV SAT1, SAT2, and SAT3 genetic signatures were mapped to the consensus sequences produced by the alignment and serotype-specific primers and probes were designed using the modified version of Primer3 2.3.7 available in Geneious (40, 41). Multiple sets of primers and probes were generated and were evaluated *in silico* to determine which primers and probes aligned to the majority of the individual sequences in the alignment. Once an rRT-PCR assay set containing two primers and a probe were identified, the nucleotides present within the sequence were evaluated against the individual sequences to determine the level of conservation across all sequences in the alignment. If a nucleotide was not conserved, a degenerate nucleotide was incorporated into the primer and/or probe to increase FMDV strain inclusivity but restricted to three degenerate nucleotides per oligomer. Primer3 2.3.7 was utilized for the *in silico* evaluation of the primer pair properties. FMDV serotype exclusivity was evaluated for all primers and probes by first utilizing BLASTn to determine that the top identifications were all the homologous serotype, followed by testing the alignment of the primers and probe against the heterologous SAT serotypes using Primer3 2.3.7 (41). The FMDV SAT serotype-specific probes were designed as dual-labeled hydrolysis TaqMan probes with a modified 5′ terminus containing a 6-carboxyfluorescein (FAM) reporter dye and a Black Hole Quencher dye (BHQ1) appended to the 3′ terminus. The SAT1, SAT2, and SAT3 serotype-specific primer and probe set sequences are listed in Table 2.

**Table 2:** FMDV SAT1, SAT2 and SAT3 serotype-specific rRT-PCR assay primers and probes.

**FMDV serotype reagent**	**FMDV SAT specific oligo name**	**Sequence (5^′^ → 3^′^)**
SAT1 forward	SAT1_3437_F	AGGCANCACACTGAYGTG
SAT1 reverse	SAT1_3502_R	GCAGRTCCAGTGTCAGTYT
SAT1 probe	SAT1_3474_P	FAM-CCTYGACCGGTTCACHCTDGT-BHQ1
SAT2 forward	SAT2_3765_F	YGTCTACAAYGGYGAGT
SAT2 reverse	SAT2_3934_R	CCKCTTCATCCKGTAGTARA
SAT2 probe	SAT2_3867_P	FAM-CGDACCCGAAGTTGAAGGTBGRCG-BHQ1
SAT3 forward	SAT3_3736_F	GYGTTGAGAMTGAAACCAC
SAT3 reverse	SAT3_ 3834_R	CWGCHCTCTTCATCCGGTA
SAT3 probe	SAT3_probe1.2	FAM-AVAGWCGCCCGAAGTTGAATGTYGTGGG-BHQ1

Characters in sequences represent degenerate bases: Y (C, T), B (C, T, G), H (C, A, T), K (G, T), M (A, C), D (A, G, T), R (A, G), W (A, T), and N (A, C, T, G).

### Total RNA extraction

Total RNA was extracted from both the FMDV cell culture isolates and tissue suspensions using the MagMAX™-96 Viral RNA Isolation Kit (AMB1836-5, Life Technologies) in combination with the MagMAX™ Express-96 Magnetic Particle Processor (Life Technologies) following the manufacturer's protocols. One microliter of VetMAX™ Xeno™ Internal Positive Control RNA (A29761, Life Technologies) was added per sample to serve as an RNA extraction control. Extractions utilized 55 μl of the sample and total RNA was eluted into 50 μl of MagMAX elution buffer (34). All RNA was stored at −70°C until evaluated by PCR or nucleic acid sequencing.

### FMDV 3D pan-serotype and SAT-specific rRT-PCR assays

Detection of pan-serotype FMDV viral genomic RNA *via* real-time reverse transcriptase PCR (rRT-PCR) was accomplished utilizing a previously published primer/probe set that detects the conserved, serotype-independent 3D RNA-dependent RNA polymerase gene of FMDV (FMDV 3D rRT-PCR) (7). The forward 1186F (5′-ACT GGGTTTTAYAAACCTGTGATG-3′) and reverse FMDV 1237R (5′-TCAACTTCTCCTKGATGGTCCCA-3′) primers amplify an 88-base-pair fragment. The FMDV dually labeled hydrolysis TaqMan probe was modified so that the 5′ end contains a 6-carboxyfluorescein (FAM) reporter dye and the 3′terminates with a Black Hole Quencher dye (BHQ1; 5′-6FAM-ATC CTC TCC TTT GCA CGC-BHQ1-3′). The Xeno internal positive control RNA was detected utilizing the proprietary VetMAX™ Xeno™ Internal Positive Control - VIC™ Assay (A29767, Applied Biosystems). Detection of pan-serotype FMDV and Xeno reactions were performed in a multiplex reaction comprised of 6.25 μl of TaqMan^®^ Fast Virus 1-Step Master Mix (4444432, Applied Biosystems), 1 μl of 25 × FMDV primers/probe mix (0.5 μM each of forward and reverse primers and 0.2 μM FAM-labeled probe), 1 μl of the VetMAX™ Xeno™ Internal Positive Control - VIC™ Assay, 5 μl RNA template topped to a final volume of 25 μl with nuclease-free H_2_O. Testing was performed on the QuantStudio™ 7 Pro Real-Time PCR System (A43183, Applied Biosystems) using a standard thermocycling program consisting of a reverse transcriptase step (50°C for 5 min), an inactivation/denaturation step (95° for 20 s) and a 45 cycle amplification step cycling between 95° for 15 s and 60° for 45 s. FMDV positive controls consisting of synthetically prepared FMDV RNA fragments amplifying at 130 base pairs and a no template negative control (NTC) composed of nuclease-free H_2_O were utilized on every run. Quantification cycle (Cq) was determined for every reaction with Cq values ≤ 35.99 considered positive for FMDV genome when accompanied by appropriate amplification curves. The Xeno reaction also adhered to the Cq cut-off of ≤ 35.99. Detection of the FMDV SAT1, SAT2, and SAT3 serotype-specific viral genomic RNA was optimized to utilize the same assay conditions and thermocycling parameters as the pan-serotype FMDV rRT-PCR with the exception that the reaction mixture contained 1.0 μM each of FMDV SAT1, SAT2, and SAT3 forward and reverse primers and 0.5 μM of the serotype-specific FAM labeled probes Table 2.

### SAT-specific rRT-PCR assay optimization and standardization

#### SAT1, SAT2 and SAT3 specific rRT-PCR assay primer/probe concentration optimization

Optimization of the concentrations of the FMDV SAT1, SAT2 and SAT3 assay primers and probes was completed by testing the ability of different reagent dilutions to detect the homologous SAT genomic RNA extracted from prototypic FMDV cell culture isolates. The FMDV cell culture isolates included SAT1/KEN/4/1998, SAT2/SAU/1/2000, and SAT3/ZIM/4/1981. Three assay conditions were examined, primers at a concentration of 2.0, 1.0, and 0.5 μM combined with the probe at a concentration of 1.0, 0.5, and 0.25 μM, respectively. All reagent concentrations were tested at a minimum in duplicate. Optimal assay performance was defined as the lowest Cq value coupled with the lowest discrepancy in Cq values between replicates with no amplification in RNA extraction and no template controls.

#### Repeatability of the SAT1, SAT2 and SAT3 rRT-PCR assays

SAT-specific rRT-PCR assay repeatability was evaluated for each assay by extracting RNA from two different prototypic FMDV cell culture isolates and performing three replicate tests on three separate days. The isolates utilized were SAT1/KEN/4/1998, SAT1/BOT/12/2006, SAT2/SAU/1/2000, SAT2/EGY/2/2012, SAT3/SAR/1/2006 and SAT3/ZAM/1/2017 as well as a PBS extraction control and a no template control (NTC). SAT1, SAT2, and SAT3 rRT-PCR assays were evaluated against both the homologous FMDV isolates as well as the heterologous SAT isolates to ensure amplification and no signal detection, respectively.

#### Analytical sensitivity of the SAT1, SAT2 and SAT3 rRT-PCR assays

Prototypic FMDV cell culture isolates representing SAT1, SAT2, and SAT3 viruses of known titer were selected to determine the analytical sensitivity using the limit of detection (LoD) for each assay. The isolates utilized were SAT1/KEN/4/1998, SAT1/KEN/121/2009, SAT2/ SAU/1/2000, SAT2/SEN/27/2009, SAT3/ZIM/4/1981 and SAT3/SAR/1/2006. Duplicate 10-fold serial dilutions of each of the FMDV isolate's cell culture supernatants from 10^0^ to at least 10^−7^ were prepared after which RNA was extracted and samples were tested using the homologous serotype SAT-specific rRT-PCR assay as well as the pan-serotype FMDV rRT-PCR assay. A standard curve was prepared from the Cq values. Assay efficiency (*E*) was calculated utilizing the formula:


$$E\;=\;-1+10^{\left(-1/slope\right)}.$$

#### Serotype specificity of the SAT1, SAT2 and SAT3 rRT-PCR assays

The analytical specificity of the SAT1, SAT2 and SAT3 rRT-PCR assays were determined by evaluating each assay against representative FMDV isolates from the A, O, Asia1 and the heterologous SAT1, SAT2 and SAT3 serotypes.

#### Analytical specificity of the SAT1, SAT2 and SAT3 rRT-PCR assays

Other vesicular disease-causing viruses were examined and included Vesicular Stomatitis Virus New Jersey Serotype (VSNJV; VS-NJ/92/CIB, VS-NJ/11/84/HBD and VS-NJ/95/COB), Vesicular Stomatitis Indiana Virus (VSIV; VS-IN/97/CRB, VS-IN/94/GUB and VS-IN/85/CLB), Swine Vesicular Disease virus (SVDV; SVD/ITL/2008, SVD/POR/1/2003, SVD/UKG/1972) and Senecavirus A (SVA; SVA prototype strain SVV-001, SVA/CAN/2015, SVA/CAN/2017).

#### Diagnostic sensitivity of the SAT1, SAT3 and SAT2 topotype VII rRT-PCR assays

Diagnostic sensitivity evaluation of the SAT1, SAT3 and SAT2 topotype VII rRT-PCR assays was conducted by utilizing SAT1, SAT2 and SAT3 FMDV cell culture isolates as representative true positive samples to test the homologous assay (see Table 3 for the list of FMDV cell culture isolates).

**Table 3:** Detection of FMDV A, O and Asia1 cell culture isolates with the serotype-specific SAT1, SAT3 and SAT2 topotype VII rRT-PCR assays.

**Serotype**	**Strain**	**SAT1 Cq**	**SAT2 Cq**	**SAT3 Cq**	**FMDV 3D Cq**	**Xeno Cq**
O	OUKG	No Cq	No Cq	No Cq	13.29	32.30
O	O1 BFS	No Cq	No Cq	No Cq	13.54	33.31
O	O1 Manisa	No Cq	No Cq	No Cq	20.14	33.63
O	O1 Campos	No Cq	No Cq	No Cq	13.54	33.57
O	O/TAN/2009	No Cq	No Cq	No Cq	12.63	33.31
O	O/CAR/2005	No Cq	No Cq	No Cq	13.88	33.29
O	O/VIT/2012	No Cq	No Cq	No Cq	12.31	33.24
O	O/LIB/2012	No Cq	No Cq	No Cq	10.75	32.88
O	O/KEN/2009	No Cq	No Cq	No Cq	13.30	33.12
O	O/NIG/2017	No Cq	No Cq	No Cq	14.78	33.97
A	A/22	No Cq	No Cq	No Cq	10.67	33.17
A	A/MAY/1997	No Cq	No Cq	No Cq	11.35	32.90
A	A/COL/1985	No Cq	No Cq	No Cq	13.52	33.29
A	A/IRN/1/1996	No Cq	No Cq	No Cq	16.69	32.81
A	A/IRN/2005	No Cq	No Cq	No Cq	16.04	33.77
A	GHA/4/1996	No Cq	No Cq	No Cq	11.67	31.41
A	BKF/4/1994	No Cq	No Cq	No Cq	10.62	31.87
A	ERI/2/1998	No Cq	No Cq	No Cq	12.50	31.65
A	ETH/6/2000	No Cq	No Cq	No Cq	11.54	31.98
A	NIG/38/2009	No Cq	No Cq	No Cq	13.81	31.41
A	ETH/12/2009	No Cq	No Cq	No Cq	13.64	32.03
A	EGY/3/2009	No Cq	No Cq	No Cq	17.85	32.00
A	SUD/1/2006	No Cq	No Cq	No Cq	12.03	31.93
A	CAR/10/2013	No Cq	No Cq	No Cq	12.67	31.56
A	NIG/A/6/2019	No Cq	No Cq	No Cq	12.91	33.15
A	NIG/A/7/2019	No Cq	No Cq	No Cq	10.58	31.85
A	NIG/A/12/2020	No Cq	No Cq	No Cq	11.87	31.43
A	NIG/A/1/2019	No Cq	No Cq	No Cq	14.78	31.74
Asia1	Asia1/Shamir/2001	No Cq	No Cq	No Cq	13.99	32.87
Asia1	Asia1/PAK/1994	No Cq	No Cq	No Cq	12.43	33.52

Samples are considered positive when the quantification cycle (Cq) is ≤ 35.99 for SAT1, SAT2, SAT3, FMDV pan-serotype 3D and Xeno control assays.

The colours used to indicate each of the Foot-and-Mouth Disease virus serotypes. Serotype O is blue, serotype A is red, serotype Asia1 is grey, serotype SAT1 is yellow, serotype SAT2 is purple and serotype SAT3 is orange. When the Cq value cells are highlighted with either yellow, purple or orange, it means that a Cq value was produced for this particular viral isolate or sample. If the yellow, purple or orange is darker and the Cq value is <35.99 then the sample was positive when evaluated by the corresponding assay. If the filled cell is the lighter version of the colour, it indicates that although a Cq value was produced, it is >35.99 and the sample is considered negative.

#### Diagnostic specificity of the SAT1, SAT3 and SAT2 topotype VII rRT-PCR assays

Evaluation of the SAT1, SAT3 and SAT2 topotype VII rRT-PCR assay diagnostic specificity was performed using samples that were confirmed to be true negatives by the FMDV 3D pan-serotype rRT-PCR. The samples were remnant negative samples obtained from previous animal experiments conducted in the laboratory (42). The samples included five tissue sample homogenates (porcine lymph node, porcine tongue and three bovine tongue tissues from different animals), five porcine serum samples, four porcine oral fluids samples, four porcine oral fluid samples and BHK-21 cell culture supernatant collected from PBS mock viral infections collected two and three DPI.

### Sequencing

#### VP1 Sanger sequencing

The VP1 gene sequence from FMDV was generated using Sanger nucleic acid sequencing based on the protocol described previously by Knowles et al. (14). Briefly, FMDV RNA was extracted and both cDNA generation and VP1 PCR amplification were accomplished using the qScript XLT One-Step RT-PCR Mastermix (95143-200, Quantabio) with FMDV serotype-specific primers. Reactions consisted of 25 μl of the 2× One-Step Toughmix, 1 μl of the 50× GelTrack Loading Dye, 0.4 mM of both the forward and reverse primer, 2 μl of 25× qScript XLT One-Step RT, 5 μl of extracted FMDV RNA and nuclease-free H_2_O to a total volume of 50 μl. VP1 FMDV cDNA amplicons were cleaned of the PCR reaction components using the QIAquick PCR Purification Kit (28104, Qiagen) while sequencing was accomplished using the BigDye^®^ Terminator v3.1 Cycle Sequencing Kit (4337452, Life Technologies) and cleaned with the DyeEx 2.0 Spin Kit (63204, Qiagen) using the manufacturer's specifications. Sequencing primers were chosen according to the recommendations for serotype described in Knowles et al. (14).

#### Next-generation sequencing

Near-full length FMDV genome sequencing was accomplished using next-generation sequencing (NGS) as previously described (43, 44). All samples were screened with the pan-serotype FMDV rRT-PCR and positives were prepared for NGS using SuperScript™ IV First-Strand Synthesis System (Life Technologies). Libraries were processed for Illumina Nextera XT sequencing and were sequenced on a MiSeq instrument using a V3 cycling kit (Illumina). Sequencing data was evaluated using a previously described workflow (43).

## Results

### Generation of specific SAT1, SAT2 and SAT3 rRT-PCR assays

The Neptune bioinformatics tool was able to identify genetic signature sequences that were highly specific for each of the SAT1, SAT2 and SAT3 FMDV serotypes (37, 45). The Neptune bioinformatics tool was able to produce these unique genetic signatures based on an inclusion group consisting of sequences from the FMDV serotype of interest and an exclusion group consisting of FMDV sequences from heterologous serotypes. The software applies a reference-based, parallelized exact-matching k-mer strategy for speed while enhancing sensitivity through allowances for inexact matches. Genetic signature identification is based on probabilistic models that derive conclusions based on statistical confidence (37). Each of the FMDV SAT1, SAT2 and SAT3 genetic signatures identified by Neptune were located in the highly variable VP1 coding region of the FMDV genome (Table 1). The signature sequence for SAT1 was predicted to be 100% specific and sensitive to SAT1 with a Neptune score of 1.0. The Neptune scores for the SAT2 and the SAT3 VP1-based signature sequences were 0.8800 and 0.8757, respectively. These genetic signatures were used as the template to discern the genomic location from which to build the SAT serotype-specific rRT-PCR assays. 286 SAT1 sequences, 378 SAT2 sequences and 50 SAT3 sequences deposited into NCBI from 2011-Fall 2021 were utilized to perform multiple alignments in Geneious (40). Primer3 (41) was used to produce SAT-specific primers and probes with non-conserved nucleotides being replaced with a degenerate nucleotide (Table 2).

### SAT-specific rRT-PCR assay optimization and standardization

#### SAT-specific rRT-PCR assay primer/probe concentration optimization

The optimal performance of the SAT1, SAT2 and SAT3 rRT-PCR assay primers and probes were determined by evaluating different concentrations of these reagents against homologous prototypic FMDV cell culture isolates (SAT1/KEN/4/1998, SAT2/SAU/1/2000 and SAT3/ZIM/4/1981). Optimal assay performance was defined as the primer/probe concentration that produced the lowest Cq with the minimal spread between technical replicate Cqs as well as no detectable amplification in the RNA extraction control nor the no template control. All extracted RNA was pre-screened with the FMDV 3D pan-serotype and Xeno rRT-PCR assays to ensure quality RNA templates (data not shown). Interestingly, the SAT1, SAT2 and SAT3 assays each performed optimally with both of the primers at a concentration of 1.0 μM and the probe at a concentration of 0.5 μM (Supplementary Figure 1).

#### Repeatability of the SAT1, SAT2 and SAT3 rRT-PCR assays

**Figure 1:**
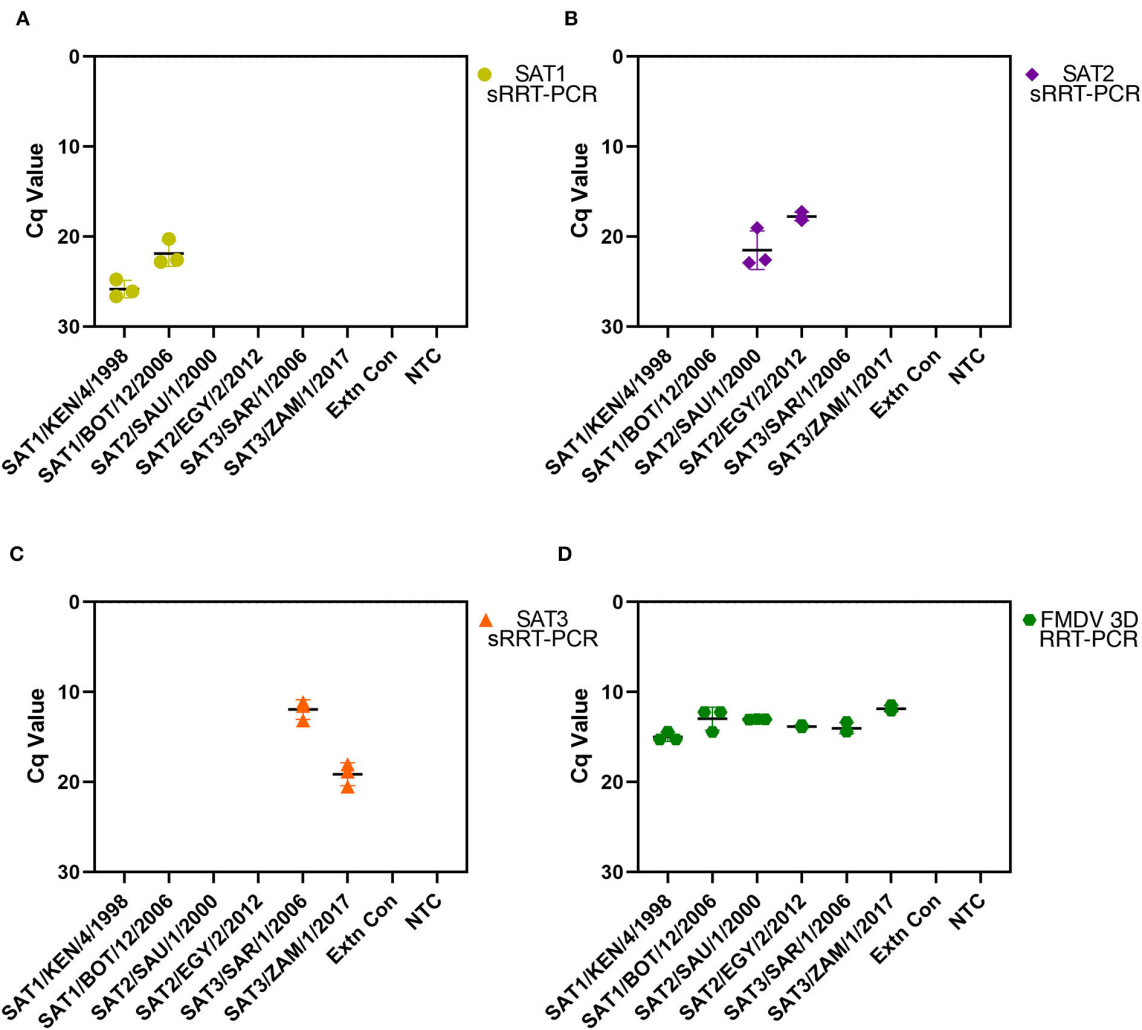
Repeatability analysis for the FMDV SAT1, SAT2 and SAT3 assays. FMDV genomic RNA was extracted on three separate days from prototypic FMDV cell culture isolates SAT1/KEN/4/1998, SAT1/BOT/12/2006, SAT2/SAU/1/2000, SAT2/EGY/2/2012, SAT3/SAR/1/2006 and SAT3/ZAM/1/2017 as well as a PBS extraction control (Extn Con). Extracted RNA from each replicate was also analyzed using the SAT1 **(A)**, SAT2 **(B)**, and SAT3 **(C)** srRT-PCR assays on three separate days. The FMDV 3D pan-serotype rRT-PCR assay (FMDV) was run in parallel with each replicate to ensure the presence of the FMDV template **(D)**. Replicate Cq values are plotted with a black line representing the mean and error bars with the corresponding FMDV serotype color (SAT1 = yellow, SAT2 = purple, SAT3 = orange and FMDV 3D pan-serotype = green).

To assess the repeatability of the SAT1, SAT2 and SAT3 rRT-PCR assays RNA was extracted from FMDV cell culture isolate prototypes (SAT1/KEN/4/1998, SAT1/BOT/12/2006, SAT2/SAU/1/2000, SAT2/EGY/2/2012, SAT3/SAR/1/2006 and SAT3/ZAM/1/2017) on three separate days followed by rRT-PCR performed also on three separate days. Each of the SAT-specific rRT-PCR assays did not amplify any of the heterologous SAT serotype RNA nor the extraction or NTC controls (Figure 1). The SAT1 rRT-PCR assay demonstrated robustness over time as the standard deviations between the three replicates for SAT1/KEN/4/1998 and SAT1/BOT/12/2006, were 0.96 and 1.41 respectively (Figure 1A). Standard deviations for the SAT2 rRT-PCR were different for the two isolates examined with the three replicates producing a standard deviation of 2.15 for SAT2/SAU/1/2000 and 0.48 for SAT2/EGY/2/2012 (Figure 1B). The SAT3 rRT-PCR assay produced standard deviations of 1.08 and 1.27 for SAT3/SAR/1/2006 and SAT3/ZAM/1/2017 (Figure 1C). Each isolate was also analyzed using the FMDV 3D pan-serotype and Xeno rRT-PCR assays to ensure that the extraction was successful and each extraction (except the negative controls) contained FMDV genomic template (Figure 1D).

#### Analytical sensitivity of the SAT1, SAT2 and SAT3 rRT-PCR assays

**Figure 2:**
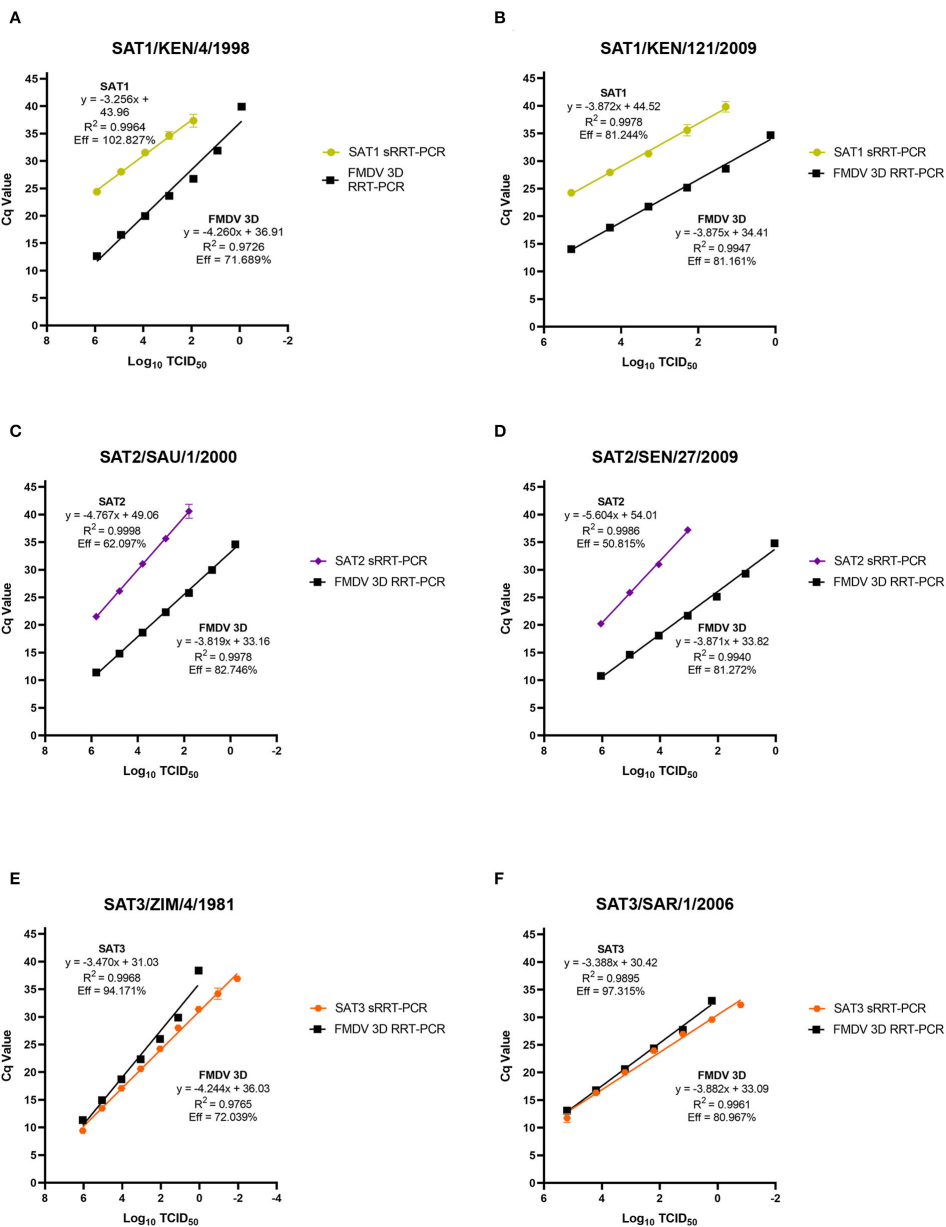
Analytical sensitivity limit of detection (LoD) analysis for the FMDV SAT1, SAT3 and SAT2 topotype VII rRT-PCR assays. FMDV genomic RNA was extracted from duplicate 10-fold serial dilutions (10^0^ – 10^−7^ or 10^−9^) of the FMDV cell culture isolates SAT1/KEN/4/1998 **(A)**, SAT1/KEN/121/2009 **(B)**, SAT2/ SAU/1/2000 **(C)**, SAT2/SEN/27/2009 **(D)**, SAT3/ZIM/4/1981 **(E)**, and SAT3/SAR/1/2006 **(F)** and were tested using the homologous serotype SAT-specific rRT-PCR assay as well as the FMDV pan-serotype 3D rRT-PCR assay. Mean Cq values are plotted with standard deviations.

The limit of detection (LoD) for the SAT1, SAT2 and SAT3 rRT-PCR assays were evaluated using prototypic FMDV cell culture isolates of a known titer. Ten-fold serial dilutions of cell culture isolated virus had the genomic RNA extracted from each of the serial dilutions and evaluated using the homologous SAT1, SAT2 and SAT3 rRT-PCR assays. There is difficulty with defining a singular LoD and rRT-PCR assay efficiency for FMDV detection as there is no all-encompassing template for a virus as genetically diverse as FMDV. As such, two representative isolates from each of the FMDV SAT serotypes were chosen (SAT1/KEN/4/1998, SAT1/KEN/121/2009, SAT2/SAU/1/2000, SAT2/SEN/27/2009, SAT3/ZIM/4/1981 and SAT3/SAR1/2006). As expected, the LoD and the PCR efficiencies of the SAT-specific rRT-PCR assays differ both with the inter-assay comparison to the 3D pan-serotype FMDV rRT-PCR and intra-assay between the two isolates tested. The LoD for the SAT1-specific rRT-PCR assay defined as the last dilution to produce a positive (Cq ≤ 35.99) signal was the 10^−3^ dilution corresponding to a detection of 10^2.92^ and 10^2.29^ TCID_50_ of the SAT1/KEN/4/1998 and SAT1/KEN/121/2009 isolates (Figures 2A,B). The efficiency of the SAT1 rRT-PCR assay was 102.827% for SAT1/KEN/4/1998 and 81.161% for SAT1/KEN/121/2009 (Figures 1A,B). In comparison, the FMDV 3D pan-serotype assay's LoD and efficiency for SAT1/KEN/4/1998 was 8.33 TCID_50_ and 71.689% and for SAT1/KEN/121/2009 it was 1.35 TCID_50_ and 81.161% (Figures 2A,B). The LoD for the SAT2 rRT-PCR assay also varied based on the isolate examined. The two isolates tested were SAT2/SAU/1/2000 and SAT2/SEN/27/2009 where the LoDs and efficiencies were 10^2.79^ TCID_50_ and 62.097% and 10^4.04^ TCID_50_ and 81.272%, respectively (Figures 2C,D). For both SAT2 isolates, the FMDV 3D pan-serotype PCR was able to detect at least four more 10-fold dilutions of each of the isolates, with estimated efficiencies ranging from 81 to 83% (Figures 2C,D). The SAT3-specific rRT-PCR assay demonstrated greater robustness with the LoDs for each of the SAT3 isolates at 0.11 TCID_50_ and 0.16 TCID_50_ for the SAT3/ZIM/4/1981 and SAT3/SAR1/2006 isolates (Figures 2E,F). The efficiencies were 94.171 and 97.315% (Figures 2E,F). For the SAT3 isolates, the FMDV 3D pan-serotype PCR had similar LoDs but the efficiencies were lower within a range of 72%−81% for the two SAT3 isolates (Figures 2E,F).

#### Serotype specificity of the SAT1, SAT2 and SAT3 rRT-PCR assays

The serotype specificity of the SAT1, SAT2, and SAT3 rRT-PCR assays were tested using a panel of FMDV isolates of known serotype. All of the isolates were screened using the FMDV 3D pan-serotype and the Xeno RNA extraction control rRT-PCR assays to ensure the RNA present was of good quality by producing Cqs of ≤ 35.99 on both assays (Tables 3, 4).

**Table 4:** Detection of FMDV SAT1, SAT2 and SAT3 cell culture isolates with the SAT1, SAT3 and SAT2 topotype VII rRT-PCR assays.

**Serotype**	**Strain**	**Topotype**	**SAT1 Cq**	**SAT2 Cq**	**SAT3 Cq**	**FMDV Cq**	**Xeno Cq**
SAT1	KEN/88/2010	I (NWZ)/–	9.37	No Cq	No Cq	13.26	33.16
SAT 1	ZAM/9/2008	I (NWZ)/–	31.95	No Cq	No Cq	14.00	33.52
SAT 1	KEN/21/2004	I (NWZ)/–	19.11	No Cq	No Cq	15.68	33.80
SAT 1	KEN/121/2009	I (NWZ)/–	23.58	No Cq	No Cq	15.38	33.96
SAT 1	KEN/24/2005	I (NWZ)/–	14.32	No Cq	No Cq	15.04	34.06
SAT 1	BOT/12/2006	III (WZ)/unnamed	22.60	No Cq	35.30	14.43	33.72
SAT 1	ETH/3/2007	IX/unnamed	No Cq	No Cq	No Cq	14.10	33.62
SAT 1	KEN/4/1998	I (NWZ)	24.77	No Cq	39.57	14.42	33.49
SAT 1	KEN/BOT/1/1968	III (WZ)	22.20	No Cq	No Cq	11.80	32.86
SAT2	ZIM/10/1991	I	No Cq	No Cq	No Cq	17.18	32.18
SAT2	ZIM/5/1981	II	No Cq	35.90	No Cq	11.21	32.37
SAT2	BOT/1/2011	III/unnamed	No Cq	No Cq	22.44	14.94	33.69
SAT 2	MOZ/1/2010	I/–	No Cq	No Cq	No Cq	13.86	33.56
SAT 2	BOT/1/2010	III/unnamed	No Cq	No Cq	No Cq	14.75	33.56
SAT 2	SUD/1/2008	XIII/–	No Cq	No Cq	No Cq	18.86	33.72
SAT 2	ZAM/8/2008	III/–	No Cq	No Cq	No Cq	14.59	33.83
SAT 2	KEN/13/2004	IV/–	No Cq	No Cq	No Cq	12.40	33.45
SAT 2	BOT/5/2009	III/unnamed	No Cq	No Cq	No Cq	14.68	33.27
SAT 2	SEN/27/2009	VII/unnamed	No Cq	23.88	No Cq	12.37	32.97
SAT 2	TAN/43/2009	IV/–	No Cq	No Cq	No Cq	14.34	33.05
SAT 2	KEN/122/2009	IV/–	No Cq	No Cq	No Cq	14.59	33.50
SAT 2	ETH/2/2007	XIII/unnamed	No Cq	No Cq	No Cq	14.09	33.69
SAT 2	KEN/2/2007	IV/–	No Cq	No Cq	No Cq	13.98	34.11
SAT2	SAU/1/2000	VII	No Cq	22.58	No Cq	13.01	33.09
SAT2	NIG/17/2017	VII	No Cq	12.71	No Cq	11.71	33.09
SAT2	NIG/18/2017	VII	No Cq	12.90	No Cq	11.78	33.87
SAT 2	EGY/2/2012	VII	No Cq	17.87	No Cq	13.70	33.56
SAT 2	NIG/PL/WAS/03/2017	VII	No Cq	15.71	36.80	12.78	34.29
SAT2	NIG/PL/PKN/01/2017	VII	No Cq	13.82	No Cq	11.97	33.86
SAT2	NIG/PL/JS/KA/1/2017	VII	No Cq	14.74	No Cq	12.36	34.27
SAT2	NIG/PL/PKN/02/2017	VII	No Cq	13.41	No Cq	13.01	34.66
SAT3	ZIM/4/1981	I (SEZ)	No Cq	No Cq	13.70	14.19	33.82
SAT 3	UGA/10/1997	V/unnamed	No Cq	No Cq	No Cq	13.25	33.60
SAT 3	SAR/1/2006	I (SEZ)/–	No Cq	37.13	11.54	13.33	34.06
SAT 3	ZIM/4/1981	I (SEZ)	No Cq	No Cq	13.02	14.95	33.47
SAT 3	SAR/1/2006	I	No Cq	No Cq	11.00	12.82	33.54
SAT 3	BEC/1/1965	II	No Cq	No Cq	21.81	14.31	33.35
SAT 3	ZAM/1/2017	II	No Cq	No Cq	18.85	11.46	33.36
SAT3	ZAM/3/2015	II	No Cq	No Cq	15.96	11.19	31.96
SAT3	ZAM/1/2017	II	No Cq	No Cq	14.33	10.07	32.25

Samples are considered positive when the Quantification cycle (Cq) is ≤ 35.99 for SAT1, SAT2, SAT3, FMDV pan-serotype FMDV 3D and Xeno control assays.

The colours used to indicate each of the Foot-and-Mouth Disease virus serotypes. Serotype O is blue, serotype A is red, serotype Asia1 is grey, serotype SAT1 is yellow, serotype SAT2 is purple and serotype SAT3 is orange. When the Cq value cells are highlighted with either yellow, purple or orange, it means that a Cq value was produced for this particular viral isolate or sample. If the yellow, purple or orange is darker and the Cq value is <35.99 then the sample was positive when evaluated by the corresponding assay. If the filled cell is the lighter version of the colour, it indicates that although a Cq value was produced, it is >35.99 and the sample is considered negative.

The SAT1, SAT2 and SAT3 rRT-PCR assays were then evaluated against FMDV serotypes O, A and Asia1 to determine specificity against non-SAT FMDV serotypes. The serotype O and A FMDV isolate panels contained FMDV isolates originating from Africa so there would be representation of viruses that co-circulate with SAT serotype viruses. All three of the SAT1, SAT2 and SAT3 rRT-PCR assays demonstrated 100% specificity against 10 serotype O isolates, 18 serotype A isolates and two Asia1 isolates (Table 3).

Next, the ability of the SAT-specific serotyping rRT-PCR assays to differentiate detection from the heterologous SAT serotypes (Table 4) was tested. The SAT1 and SAT2 specific rRT-PCR assays demonstrated 100% specificity as no cross-reaction with any of the heterologous SAT1, SAT2 nor SAT3 viruses were observed (Table 4).

While no cross-reactivity was observed when the SAT3 assay was evaluated against any of the SAT2 isolates, one of the nine SAT1 isolates was identified as weakly positive. The SAT3 assay produced a Cq value of 35.30 with the SAT1/BOT/12/2006 isolate, a topotype III virus (Table 4). However, the SAT1-specific rRT-PCR assay produced a Cq of 22.60, with that isolate, a Cq value that was 12.70 Cqs lower than that SAT3 assay, correctly identifying the isolate as a SAT1 virus (Table 4). The SAT3-specific assay also demonstrated cross-reactivity with only one of the 22 SAT2 isolates. A Cq of 22.44 was produced when the SAT3 assay was tested against the SAT2/BOT/1/2011 isolate. A nucleic acid sequence for SAT2/BOT/1/2011 was not available on NCBI, so the VP1 coding sequence of the isolate was produced *via* Sanger sequencing. Alignment of the primers and probes to the Sanger-produced SAT2/BOT/1/2011, VP1 sequence revealed that only the reverse primer aligned but, not the forward primer nor the probe.

#### Analytical specificity of the SAT1, SAT2 and SAT3 rRT-PCR assays

The classic lesions produced by FMDV infection cannot be distinguished clinically from other vesicular disease-causing viruses and a definitive diagnosis is obtained through laboratory diagnostic testing. As such, the analytical specificity of each of the SAT1, SAT2 and SAT3 rRT-PCR assays was evaluated using three cell culture viral isolates representing VSNJV, VS-IV, SVDV and SVA. No detectable amplification was produced by any of the SAT-specific rRT-PCR assays when tested against templates from these vesicular viruses (data not shown).

#### Diagnostic sensitivity of SAT1, SAT3 and SAT2, topotype VII rRT-PCR assays

Next, the diagnostic sensitivity of the SAT-specific assays was evaluated by testing the ability of the SAT-specific serotyping rRT-PCR assays to detect the homologous SAT viral cell culture isolates. The nine SAT1 isolates included topotype I, III and IX viruses, 22 SAT2 isolates that included topotypes I–IV, VII and XIII and nine SAT3 isolates that included topotypes I, II and V (Table 4).

The SAT1 assay was able to detect eight out of the nine FMDV SAT1 isolates with Cqs ranging from 9.37 to 31.95 (Table 4). As such, the diagnostic sensitivity of the assay was 88.89% (Supplementary Table 1). The SAT1 assay failed to detect the SAT1/ETH/3/2007 isolate, a topotype IX virus. An alignment between the SAT1 assay primers and probe and the SAT1/ETH/3/2007 VP1 nucleic acid sequence (accession number: FJ798154.1) was performed in Geneious (40). This *in silico* analysis revealed that neither the forward nor reverse primer were able to bind the sequence and that there were two mismatches in the probe alignment (data not shown).

Testing of the SAT2-specific assay against 22 different SAT2 isolates revealed that the assay had topotype VII specificity (Table 4). No detectable fluorescence was produced from any of the SAT2, topotype I, III, IV nor XIII isolates. One SAT2, topotype II virus, SAT2/ZIM/5/1981, was identified as a very weak positive with a Cq of 35.90 (Table 4). As the SAT2-specific rRT-PCR assay demonstrated specificity for only the topotype VII isolates, the assay was redefined as the SAT2, topotype VII-specific rRT-PCR assay. The diagnostic sensitivity calculated from the nine FMDV SAT2 topotype VII isolates was 100% (Supplementary Table 1).

The SAT3-specific rRT-PCR assay was able to detect eight out of the nine SAT3 isolates with Cqs ranging from 11.00 to 21.80 (Table 4). From these nine FMDV SAT3 isolates the diagnostic sensitivity was 88.89% (Supplementary Table 1). SAT3/UGA/10/1997 was the only SAT3 isolate that the assay failed to detect, and was the only topotype V virus tested. An alignment of the SAT3-specific primers and probe with SAT3/UGA/10/97 (accession number: KY091308.1) revealed that the SAT3 probe was capable of binding to the target nucleic acid sequence, but neither the forward nor reverse primers were (data not shown).

#### Diagnostic specificity of SAT1, SAT3 and SAT2, topotype VII rRT-PCR assays

Diagnostic specificity of the SAT1, SAT3 and SAT2, topotype VII rRT-PCR assays was investigated utilizing FMDV negative samples. All negative samples were defined as such by no amplification produced when tested by the FMDV 3D pan-serotype rRT-PCR but also the presence of a quality template by producing a Cq ≤ 35.99 on the Xeno rRT-PCR assay. Eighteen remnant clinical samples (42) and two mock viral infections were evaluated. These samples included five tissue sample homogenates (porcine lymph node, porcine tongue and three bovine tongue tissues from different animals), five porcine serum samples, four porcine oral swab samples, four porcine oral fluid samples and BHK-21 cell culture supernatant collected from PBS mock viral infections collected two and three DPI. Each of the SAT1, SAT3 and SAT2, topotype VII rRT-PCR assays did not produce any detectable fluorescent signal and thus the diagnostic specificity of these assays was 100% (Supplementary Table 1).

### Detection and serotyping of FMDV samples from Nigeria

The detection and differentiation abilities of the SAT1, SAT3 and SAT2 topotype VII rRT-PCR assays were evaluated using bovine tissue samples collected from Nigeria. The 99 tissue samples were collected from various states in Nigeria in 2020. Serotyping of the samples was accomplished utilizing Illumina-Nextera NGS sequencing. Produced sequences had to contain the VP1 coding sequence and the produced contigs were searched against the BLASTn database to obtain the closest serotype match. Of the 99 samples, NGS identified 63 as serotype O/EA3, 12 as A/WAG/IV and 24 as SAT2/VII (Table 5). Samples were organized based on the area collected. All samples were tested using the FMDV pan-serotype 3D rRT-PCR and Xeno RNA extraction control RTT-PCR assays and found to be positive on both. The SAT1-specific rRT-PCR assay demonstrated 100% specificity as the assay produced no Cqs for any of the 99 samples (Table 5). The SAT3-specific rRT-PCR only incorrectly identified one of the 99 samples as positive for SAT3. However, this sample, SAT2/NIG/PL/JA/2/2020, was also identified correctly by the SAT2, topotype VII specific rRT-PCR with a Cq of 18.87 vs. the Cq of 35.56 produced by the SAT3 assay that was also just below the positive cutoff (Table 5). The SAT2, topotype VII specific rRT-PCR demonstrated the assay sensitivity to be 100% in that it correctly identified all 24 SAT2 samples (Table 5). This assay demonstrated no cross-reactivity with any of the samples identified as A/WAG/IV viruses. However, some cross-reactivity was demonstrated by the SAT2, topotype VII specific rRT-PCR assay where five of the 63 samples identified as O/EA3 viruses were positive on the SAT2, topotype VII assay (Table 5). Interestingly, since the tissue samples were collected in the same year and close in proximity, it can't be ruled out that these samples may contain SAT2/VII genomic material.

**Table 5:** Detection of FMDV from bovine tissue samples collected from Nigeria using the SAT1, SAT3 and SAT2 topotype VII rRT-PCR assays.

**Isolate**	**Serotype/Topotype/Lineage**	**SAT1 Cq**	**SAT2 Cq**	**SAT3 Cq**	**FMDV 3D Cq**	**Xeno Cq**
O/NIG/BAU/BAU/1/2020	O/EA3	No Cq	No Cq	No Cq	17.96	31.36
O/NIG/BAU/BAU/5/2020	O/EA3	No Cq	No Cq	No Cq	23.12	31.59
O/NIG/BAU/BAU/13/2020	O/EA3	No Cq	No Cq	No Cq	24.04	31.11
O/NIG/BAU/BAU/14/2020	O/EA3	No Cq	No Cq	No Cq	21.33	31.44
O/NIG/BAU/BAU/17/2020	O/EA3	No Cq	No Cq	No Cq	25.77	31.83
O/NIG/BAU/BAU/21/2020	O/EA3	No Cq	No Cq	37.81	16.22	31.53
O/NIG/BAU/BAU/22/2020	O/EA3	No Cq	No Cq	No Cq	15.73	32.14
SAT2/NIG/PL/BLD/1/2020	SAT2/VII	No Cq	19.57	No Cq	13.73	31.82
SAT2/NIG/PL/BLD/2/2020	SAT2/VII	No Cq	21.01	No Cq	15.32	31.63
SAT2/NIG/PL/BLD/3/2020	SAT2/VII	No Cq	14.84	No Cq	9.82	31.41
SAT2/NIG/PL/BLD/5/2020	SAT2/VII	No Cq	16.63	No Cq	11.80	31.04
SAT2/NIG/PL/BLD/6/2020	SAT2/VII	No Cq	22.26	No Cq	15.79	31.20
SAT2/NIG/PL/BLD/7/2020	SAT2/VII	No Cq	21.25	No Cq	15.22	30.99
SAT2/NIG/PL/BLD/8/2020	SAT2/VII	No Cq	14.69	No Cq	10.34	31.37
O/NIG/PL/BLD/9/2020	O/EA3	No Cq	No Cq	No Cq	26.64	31.72
O/NIG/KN/BMF/1/2020	O/EA3	No Cq	No Cq	No Cq	19.92	31.46
O/NIG/KN/BMF/2/2020	O/EA3	No Cq	No Cq	No Cq	15.76	32.18
O/NIG/KN/BMF/3/2020	O/EA3	No Cq	No Cq	No Cq	17.78	31.60
O/NIG/KN/BMF/4/2020	O/EA3	No Cq	No Cq	36.13	18.17	31.50
O/NIG/KN/BMF/5/2020	O/EA3	No Cq	No Cq	No Cq	27.26	31.94
O/NIG/KN/RMG/1/2020	O/EA3	No Cq	No Cq	No Cq	18.34	31.46
O/NIG/PL/BK/1/2020	O/EA3	No Cq	No Cq	No Cq	16.77	31.29
SAT2/NIG/PL/BK/2/	SAT2/VII	No Cq	28.90	No Cq	22.17	30.84
O/NIG/PL/BK/3/2020	O/EA3	No Cq	No Cq	No Cq	18.08	31.00
O/NIG/PL/BK/4/2020	O/EA3	No Cq	No Cq	No Cq	20.78	31.37
A/NIG/PL/BK/5/2020	A/WAG/IV	No Cq	No Cq	No Cq	13.85	32.71
O/NIG/PL/BK/6/2020	O/EA3	No Cq	No Cq	No Cq	23.23	31.77
A/NIG/PL/BK/7/2020	A/WAG/IV	No Cq	No Cq	No Cq	17.21	32.21
SAT2/NIG/PL/BK/8/2020	SAT2/VII	No Cq	28.62	No Cq	21.72	30.60
O/NIG/PL/BK/31/2020	O/EA3	No Cq	No Cq	No Cq	25.14	32.42
O/NIG/PL/BK/32/2020	O/EA3	No Cq	No Cq	No Cq	25.43	32.05
O/NIG/PL/BK/33/2020	O/EA3	No Cq	No Cq	No Cq	23.00	32.31
O/NIG/KD/ZA/1/2020	O/EA3	No Cq	No Cq	No Cq	19.77	30.67
O/NIG/KD/ZA/2/2020	O/EA3	No Cq	43.16	No Cq	15.72	30.70
O/NIG/KD/ZA/4/2020	O/EA3	No Cq	No Cq	No Cq	24.98	30.63
O/NIG/KD/ZA/5/2020	O/EA3	No Cq	No Cq	No Cq	14.16	31.73
O/NIG/KN/KN/1/2020	O/EA3	No Cq	No Cq	No Cq	18.37	30.94
O/NIG/KN/KN/2/2020	O/EA3	No Cq	No Cq	No Cq	17.47	30.81
SAT2/NIG/PL/JS/1/2020	SAT2/VII	No Cq	19.75	No Cq	14.17	31.26
SAT2/NIG/PL/JS/2/2020	SAT2/VII	No Cq	18.87	35.56	13.69	31.32
O/NIG/KT/KT/1/2020	O/EA3	No Cq	No Cq	No Cq	17.02	30.70
O/NIG/KT/KT/2/2020	O/EA3	No Cq	No Cq	No Cq	20.58	31.38
O/NIG/KT/KT/3/2020	O/EA3	No Cq	No Cq	No Cq	13.78	32.19
O/NIG/BAU/TR/1/2020	O/EA3	No Cq	No Cq	No Cq	19.10	31.70
O/NIG/PL/RY/1/2020	O/EA3	No Cq	27.13	No Cq	24.91	31.63
SAT2/NIG/PL/RY/2/2020	SAT2/VII	No Cq	17.09	No Cq	15.79	31.49
SAT2/NIG/PL/RY/3/2020	SAT2/VII	No Cq	28.58	No Cq	21.78	31.00
O/NIG/KD/KD/1/2020	O/EA3	No Cq	No Cq	No Cq	21.86	31.22
O/NIG/KD/KD/2/2020	O/EA3	No Cq	No Cq	No Cq	21.04	30.89
O/NIG/KD/KD/3/2020	O/EA3	No Cq	No Cq	No Cq	15.74	31.35
O/NIG/KD/KD/4/2020	O/EA3	No Cq	No Cq	No Cq	16.86	31.56
O/NIG/KD/KD/5/2020	O/EA3	No Cq	No Cq	No Cq	14.70	31.42
A/NIG/KD/KGR/1/2020	A/WAG/IV	No Cq	No Cq	No Cq	23.04	31.92
A/NIG/KD/KGR/2/2020	A/WAG/IV	No Cq	No Cq	No Cq	22.21	31.98
A/NIG/KD/KGR/3/2020	A/WAG/IV	No Cq	No Cq	No Cq	11.91	32.07
A/NIG/KD/KGR/4/2020	A/WAG/IV	No Cq	No Cq	No Cq	26.40	31.90
A/NIG/KD/KGR/7/2020	A/WAG/IV	No Cq	No Cq	No Cq	17.28	32.06
A/NIG/KD/KGR/8/2020	A/WAG/IV	No Cq	No Cq	No Cq	16.31	31.71
A/NIG/KD/KGR/9/2020	A/WAG/IV	No Cq	No Cq	No Cq	23.03	31.59
A/NIG/KD/KGR/10/2020	A/WAG/IV	No Cq	No Cq	No Cq	18.33	31.26
A/NIG/KD/KGR/11/2020	A/WAG/IV	No Cq	No Cq	No Cq	21.92	32.27
A/NIG/KD/KGR/14/2020	A/WAG/IV	No Cq	No Cq	No Cq	21.68	32.11
O/NIG/PL/KAN/1/2020	O/EA3	No Cq	No Cq	No Cq	18.67	32.88
O/NIG/PL/KAN/2/2020	O/EA3	No Cq	No Cq	No Cq	14.07	32.48
O/NIG/PL/KAN/3/2020	O/EA3	No Cq	No Cq	No Cq	15.12	32.35
O/NIG/PL/KAN/4/2020	O/EA3	No Cq	No Cq	No Cq	12.61	32.37
O/NIG/PL/KAN/5/2020	O/EA3	No Cq	No Cq	No Cq	14.17	32.28
O/NIG/PL/KAN/6/2020	O/EA3	No Cq	No Cq	No Cq	15.89	31.84
O/NIG/AD/GMB/2/2020	O/EA3	No Cq	No Cq	No Cq	19.98	32.24
O/NIG/AD/GMB/3/2020	O/EA3	No Cq	No Cq	No Cq	24.28	31.49
O/NIG/AD/GMB/4/2020	O/EA3	No Cq	No Cq	No Cq	23.79	31.99
O/NIG/AD/GMB/5/2020	O/EA3	No Cq	No Cq	No Cq	22.12	31.97
O/NIG/PL/KA/1/2020	O/EA3	No Cq	No Cq	No Cq	16.53	32.39
O/NIG/PL/KA/2/2020	O/EA3	No Cq	No Cq	No Cq	16.60	32.58
SAT2/NIG/PL/BL/1/2020	SAT2/VII	No Cq	22.44	No Cq	16.46	33.89
SAT2/NIG/PL/BL/2/2020	SAT2/VII	No Cq	13.11	No Cq	10.17	32.56
SAT2/NIG/PL/BL//3/2020	SAT2/VII	No Cq	15.71	No Cq	11.45	32.82
SAT2/NIG/PL/BL/4/2020	SAT2/VII	No Cq	19.22	No Cq	14.75	33.54
SAT2/NIG/PL/BL/6/2020	SAT2/VII	No Cq	14.02	No Cq	12.19	32.70
SAT2/NIG/PL/BL/7/2020	SAT2/VII	No Cq	16.	No Cq	12.03	32.90
SAT2/NIG/PL/BL/8/202	SAT2/VII	No Cq	19.95	No Cq	15.62	32.45
SAT2/NIG/PL/BL/9/2020	SAT2/VII	No Cq	22.35	No Cq	16.75	32.29
SAT2/NIG/PL/BL/10/2020	SAT2/VII	No Cq	15.67	No Cq	11.64	32.97
SAT2/NIG/PL/BL/11/2020	SAT2/VII	No Cq	12.81	No Cq	9.92	32.97
SAT2/NIG/PL/BL/12/2020	SAT2/VII	No Cq	24.16	No Cq	19.50	32.76
O/NIG/PL/JE/13/2020	O/EA3	No Cq	34.90	No Cq	28.71	33.25
O/NIG/PL/JE/16/2020	O/EA3	No Cq	35.56	No Cq	29.15	32.76
O/NIG/PL/JE/17/2020	O/EA3	No Cq	No Cq	No Cq	15.51	33.26
O/NIG/PL/JE/19/2020	O/EA3	No Cq	38.54	No Cq	15.42	32.29
O/NIG/PL/JN/20/2020	O/EA3	No Cq	40.46	No Cq	20.96	32.11
O/NIG/PL/JN/21/2020	O/EA3	No Cq	30.87	No Cq	18.87	32.11
O/NIG/PL/JN/22/2020	O/EA3	No Cq	No Cq	No Cq	24.12	31.80
O/NIG/PL/JN/23/2020	O/EA3	No Cq	No Cq	No Cq	22.48	32.55
O/NIG/PL/JN/24/2020	O/EA3	No Cq	30.11	No Cq	16.45	32.31
O/NIG/PL/JN/25/2020	O/EA3	No Cq	No Cq	No Cq	23.03	32.10
O/NIG/PL/JN/26/2020	O/EA3	No Cq	No Cq	No Cq	23.87	31.97
O/NIG/PL/JN/27/2020	O/EA3	No Cq	No Cq	No Cq	19.80	32.13
O/NIG/PL/JN/28/2020	O/EA3	No Cq	33.30	No Cq	18.69	32.08
O/NIG/PL/JN/29/2020	O/EA3	No Cq	No Cq	No Cq	23.24	31.19

Next-generation sequencing was utilized to identify FMDV serotype. Samples are considered positive when the quantification cycle (Cq) is ≤ 35.99 for SAT1, SAT2, topotype VII, SAT3, FMDV pan-serotype 3D and Xeno control assays.

The colours used to indicate each of the Foot-and-Mouth Disease virus serotypes. Serotype O is blue, serotype A is red, serotype Asia1 is grey, serotype SAT1 is yellow, serotype SAT2 is purple and serotype SAT3 is orange. When the Cq value cells are highlighted with either yellow, purple or orange, it means that a Cq value was produced for this particular viral isolate or sample. If the yellow, purple or orange is darker and the Cq value is <35.99 then the sample was positive when evaluated by the corresponding assay. If the filled cell is the lighter version of the colour, it indicates that although a Cq value was produced, it is >35.99 and the sample is considered negative.

## Discussion

FMDV is one of the most economically devastating pathogens worldwide leading afflicted areas to suffer both direct and indirect losses. For many countries elimination of FMDV is through a strict stamp-out policy leading to mass animal culling. Other nations have controlled the disease through the implementation of a vaccine policy. Due to the virus' highly contagious nature, incursions are feared by all nations. The paramount method to combat FMDV spread is preparedness. This is accomplished at the laboratory level by the establishment of rapid, sensitive, and specific diagnostic tools.

Currently, the most sensitive validated first-line FMDV diagnostic assays are serotype-independent, able to define the presence of the virus in a sample. After FMDV is detected it is important to identify which of the seven antigenically distinct serotypes the virus belongs to. This is because FMDV serotyping is the crucial first step in establishing a VP1 Sanger-based sequencing plan and identifying an appropriate vaccine match. Currently, the most commonly used method for FMDV serotyping is the Ag-ELISA. However, with a sensitivity as low as 80%, it often requires isolation of the virus to produce enough analyte for detection and requires an overnight incubation step (3, 10, 11). Sanger sequencing of the FMDV VP1 coding region also requires prior knowledge of viral serotype to select appropriate amplification and sequencing primers (14). rRT-PCR methodologies represent an attractive method to facilitate FMDV serotyping due to its lower resource and technical requirements. This can only be accomplished if genetic signatures that are both unique to the FMDV serotype and all-encompassing to the intra-serotype strains are identified, a task that is quite difficult given the extreme genetic diversity of FMDV.

The Neptune bioinformatics tool was developed to identify genetic signature sequences that are conserved within a defined inclusion group yet absent from defined exclusion groups (37). In this study, Neptune was used for the first time to identify genetic sequences unique to a viral serotype, specifically the FMDV SAT1, SAT2 and SAT3 serotypes. The Neptune-generated SAT1, SAT2 and SAT3 genetic signatures were all predicted to have high sensitivity and specificity generating scores >87%. Each of these SAT serotype-specific genetic signatures mapped to the VP1 coding regions of the FMDV genome. This is not surprising, as the VP1 coding locus displays the greatest amount of diversity with ~30%−50% discrepancy between serotypes (46). As such, SAT serotype-specific-TaqMan-based rRT-PCR assays were designed within the Neptune-generated signature sequences.

However, the extent of FMDV's genetic diversity extends beyond viral serotype and down into viral strains/subtypes. To facilitate the design of SAT-specific primers and probes, SAT1, SAT2 and SAT3 viral sequences published between 2011 and Fall 2021 were retrieved from NCBI and aligned to ascertain the level of genetic conservation. As foreseeable, there was significant nucleotide level diversity displayed by the intra-serotype strain/subtype sequence alignments. To reconcile these discrepancies, degenerative nucleotides were incorporated into the primers and probes to expand genetic coverage in an attempt to increase the intra-serotype sensitivity of the assay. However, no more than three degenerative nucleotides were incorporated into any of the oligonucleotides to not sacrifice assay specificity. While there is no defined limit of degenerate nucleotides in a primer or probe, expansive usage can lead to decreased assay specificity and the potential for the assay oligonucleotides to self-anneal or bind to each other. As such, successful rRT-PCR assay generation with incorporated degenerative nucleotides in the primers and/or probes must be tested empirically as was done in this study.

Repeatability analysis revealed the robustness of the SAT1, SAT2 and SAT3 rRT-PCR assays. Triplicate independent RNA extractions and rRT-PCR analysis revealed the resilience of each of the SAT-specific assays as the standard deviations produced were at most 2.15 (Figure 1).

The analytical sensitivity, LoD testing of the SAT-specific rRT-PCR assays revealed that the dynamic range was the greatest with the SAT3 rRT-PCR assay, followed by the SAT1 and then the SAT2 topotype VII (Figure 2). It is difficult to report singular assay specificity when the genetic diversity of the analyte is so great. Typically, DNA–based plasmids are utilized for analytical sensitivity analysis, however, they do not control for the variability introduced from the RNA extraction and reverse transcriptase processes that are integral upstream rRT-PCR assay procedures. As such, this study utilized FMDV isolates that were serially diluted prior to RNA extraction to account for those variables. As expected, utilizing this model for analytical sensitivity testing demonstrated the variability in the LoD and the rRT-PCR efficiency both within and between the different SAT-specific and FMDV pan-serotype 3D assays. Nonetheless, all three of the SAT-specific assays demonstrated that there was sufficient dynamic range to detect a variety of FMDV strains albeit less than the FMDV pan-serotype 3D rRT-PCR assay (Figure 2). Despite differences in assay robustness, the intention of the SAT-specific assays are to be used to evaluate samples that had been previously identified as positive by the FMDV pan-serotype 3D assay, as a way to identify serotype, not as a first-line diagnostic tool.

Genetic *in silico* predictive methodologies provide an appropriate starting point when defining genetic signatures. However, they are only as good as the data that is supplied to them and there is potential for them not to translate into viable *in vitro* reagents. Fortunately, that was not the case with the Neptune-based, SAT-specific rRT-PCR assays as the serotype specificity of each assay was 100% when tested against heterologous serotype O, A and Asia1 viruses (Table 3).

The serotype specificity of the SAT-specific rRT-PCR assays against heterologous SAT serotypes was 100% for SAT1 and SAT2 assays (Table 4). Though the SAT3 rRT-PCR demonstrated cross-reactivity with one of the nine SAT1 isolates, SAT1/BOT/12/2006, the Cq value for the SAT1/BOT/12/2006 produced by the SAT1-specific assay was 22.60 as opposed to the 35.30 produced by the SAT3 assay. Since the SAT-specific rRT-PCR assays are intended to be utilized in tandem on a single sample, the lower Cq value produced would define the viral serotype and therefore, SAT1/BOT/12/2006 would be serotyped as a SAT1 virus. Each of the SAT-specific rRT-PCR assays also demonstrated 100% analytical specificity when tested against VSNJV, VSIV, SVDV, and SVA. It should be noted that the intended use of the serotyping assays would be secondary after the detection of FMDV genomic RNA by the pan-serotype 3D rRT-PCR and exclusion of vesicular disease differentials.

When the diagnostic sensitivity of the assays was evaluated, both the SAT1 and SAT3-specific assays were able to detect eight out of the nine viruses of a homologous serotype. The SAT1/ETH/3/2007 isolate (accession number: FJ798154.1) not detected by SAT1 rRT-PCR assay failed to bind the SAT1 primers and probe when the sequences were aligned. The SAT1/ETH/3/2007 was the only SAT1, topotype IX virus that the SAT1 rRT-PCR was evaluated against. Therefore, the assay may have limitations for that particular topotype or specifically this viral isolate given that it was submitted to NCBI prior to the 2011 cut-off used for assay oligonucleotide design. The SAT3-specific rRT-PCR assay also failed to detect one of the SAT3 isolates, SAT3/UGA/10/1997. This is likely since the sequences retrieved from NCBI to build the SAT3 alignments were limited to a 10-year window (2011-Fall 2021). This was done to design primers and probes that were likely to bind to currently circulating FMDVs.

Interestingly, when the SAT2-specific rRT-PCR assay was evaluated against the panel of 22 SAT2 isolates, a topotype VII specificity was revealed. This was not the original intention of the assay, but of the 22 SAT2 isolates examined, eight were topotype VII viruses and were all detected with Cqs ranging from 12.71 to 23.88, demonstrating a strong topotype VII specificity (Table 4). As such, the assay was redefined as the SAT2, topotype VII specific rRT-PCR assay. It has been noted previously that SAT2 viruses exhibit high genetic intra-serotype diversity within their VP1 sequences diverging by ~20% (47). SAT2 topotype VII viruses also have the furthest geographic distribution in comparison to the other SAT2 topotypes encompassing most of the Northern part of Africa (48). It is plausible that SAT2 topotype VII viruses are retrieved more frequently resulting in sequences being reported more than the other topotypes, thus leading to an overabundance of SAT2 topotype VII sequences in the NCBI database.

Diagnostic specificity of SAT1, SAT3 and SAT2 topotype VII rRT-PCR assays was revealed to be 100% when evaluated against 18 negative clinical samples and two mock viral infections. These results support that there is no off-target amplification of host nucleic acid and that only when there is the presence of the specific FMDV SAT genome, there is template detection.

The SAT-specific rRT-PCR assays were evaluated against bovine tissue samples collected from Nigeria (Table 5). This was to determine assay utility with true clinical veterinary samples without a prior isolation step to mimic a clinical diagnostic scenario. All samples were screened utilizing the FMDV pan-serotype 3D rRT-PCR to ensure the presence of the FMDV genome, and viral serotyping was accomplished utilizing NGS. The SAT-specific rRT-PCR assays retained high sensitivity and specificity for SAT2 topotype VII, correctly identifying 100% of the SAT2 topotype VII samples and potentially cross-reacting with only five of 63 serotype O samples. The O-specific bovine tissue samples were collected from the same location as SAT2 samples. As such, these results may be true positive for SAT2 potentially due to a mixed infection or cross-contamination during sample collection. The SAT1 and SAT3-specific rRT-PCR assay also demonstrated high analytical specificity for field samples. The only sample producing a positive Cq value for SAT3 produced a much lower Cq value with the SAT2-topotype VII rRT-PCR assay, thus, correctly identifying the sample as a SAT2, topotype VII virus.

To the best of our knowledge, this study was the first to utilize the novel Neptune bioinformatics tool to generate unique FMDV serotype signatures to build rRT-PCR assays. Interestingly, Neptune produced serotype-specific signatures in the VP1 locus of the FMDV genome. Previous FMDV rRT-serotyping assays also exploited the diversity of the VP1 coding region to design primers and probes. However, due to the vast genetic diversity of FMDV, most of the current rRT-PCR serotyping assays target geographically distinct lineages. Bachanek-Bankowska et al., 2016 developed sensitive and specific TaqMan-based rRT-PCR assays for the detection of A/AFRICA/G-I, O/EA-2, O/EA-4, SAT1/I and SAT2/IV FMDVs circulating in East Africa (27). rRT-PCR capable of detecting and distinguishing serotype O, A and Asia1 serotypes circulating in West Eurasia and the Middle East have also demonstrated serotyping utility (25, 26). Most recently, Lim et al. (32) were able to serotype O, A, and Asia viruses with a VP1-directed rRT-PCR evaluated against Asian FMDV isolates (32). The SAT1 and SAT3-specific rRT-PCR assays tested in this study appear to have no preference for topotype or lineage, with the caveat that testing multiple lineages would need to be expanded but was restricted due to viral isolate availability. Unexpectedly, the SAT2-specific rRT-PCR assay designed in this study resulted in an assay with SAT2, topotype VII tropism. As such, the design of FMDV rRT-PCR that are intentionally geographically restricted continues to represent the most viable method to utilize rRT-PCR to serotype FMDV. Even so, with the dynamic evolutionary nature of circulating FMDVs, it is crucial to continue to update genomic databases and continue to evaluate these assays against contemporary strains. It is likely that over time new strains/subtypes and the introduction of mutations by an RNA-dependent-RNA polymerase will demand that additional primers and/or probes be added to the current SAT1, SAT3 and SAT2, topotype VII rRT-PCR assays presented in this work.

## Data availability statement

The raw data supporting the conclusions of this article will be made available by the authors, without undue reservation.

## Author contributions

Conceptualization: CN, OL, and TC. Methodology and writing—review and editing: TC, CN, OL, KH, HU, MN, and PS. Validation: TC, PS, MN, and KH. Formal analysis: TC, CN, PS, and MN. Investigation, data curation, and supervision: TC and CN. Writing—original draft preparation: TC. Project administration: CN. Funding acquisition: CN and OL. All authors have read and agreed to the published version of the manuscript.

## Funding

This project was funded by the Canadian Food Inspection Agency Technology Development fund (TD N-000184).

## Conflict of interest

The authors declare that the research was conducted in the absence of any commercial or financial relationships that could be construed as a potential conflict of interest.

## Publisher's note

All claims expressed in this article are solely those of the authors and do not necessarily represent those of their affiliated organizations, or those of the publisher, the editors and the reviewers. Any product that may be evaluated in this article, or claim that may be made by its manufacturer, is not guaranteed or endorsed by the publisher.
