# Supplementary material for: Development of reverse-transcriptase, real-time PCR assays to distinguish the Southern African Territories (SAT) serotypes 1 and 3 and topotype VII of SAT2 of Foot-and-Mouth Disease Virus
Source: Front Vet Sci. 2022 Sep 20;9:977761. doi: 10.3389/fvets.2022.977761 (PMC9530708; doi:10.3389/fvets.2022.977761)
Supplement: Supplementary file 1 [file Table_1.DOCX]

**Supplemental Table 1**: Diagnostic sensitivity and specificity of the serotype-specific SAT1, SAT3 and SAT2 topotype VII rRT-PCR assays. Note: FMDV genome presence in the known positive samples or absence in the known negative samples was confirmed by FMDV 3D rRT-PCR. Known positive samples were all positive by the FMDV 3D rRT-PCR. Serotype-specific reference positives were confirmed by genome sequencing.

| **SAT1 rRT-PCR Assay** | | | |
| --- | --- | --- | --- |
|  |  | Known Positives | Known Negatives |
| Test Results | Positive | 8 | 0 |
|  | Negative | 1 | 20 |
|  |  | **Diagnostic Sensitivity** | **Diagnostic Specificity** |
|  |  | 88.89% | 100% |
| **SAT2 topotype VII rRT-PCR Assay** | | | |
|  |  | Known Positives | Known Negatives |
| Test Results | Positive | 9 | 0 |
|  | Negative | 0 | 20 |
|  |  | **Diagnostic Sensitivity** | **Diagnostic Specificity** |
|  |  | 100% | 100% |
| **SAT3 rRT-PCR Assay** | | | |
|  |  | Known Positives | Known Negatives |
| Test Results | Positive | 8 | 0 |
|  | Negative | 1 | 20 |
|  |  | **Diagnostic Sensitivity** | **Diagnostic Specificity** |
|  |  | 88.89% | 100% |
