# Supplementary figures and images for: Development of reverse-transcriptase, real-time PCR assays to distinguish the Southern African Territories (SAT) serotypes 1 and 3 and topotype VII of SAT2 of Foot-and-Mouth Disease Virus
Source: Front Vet Sci. 2022 Sep 20;9:977761. doi: 10.3389/fvets.2022.977761 (PMC9530708; doi:10.3389/fvets.2022.977761)

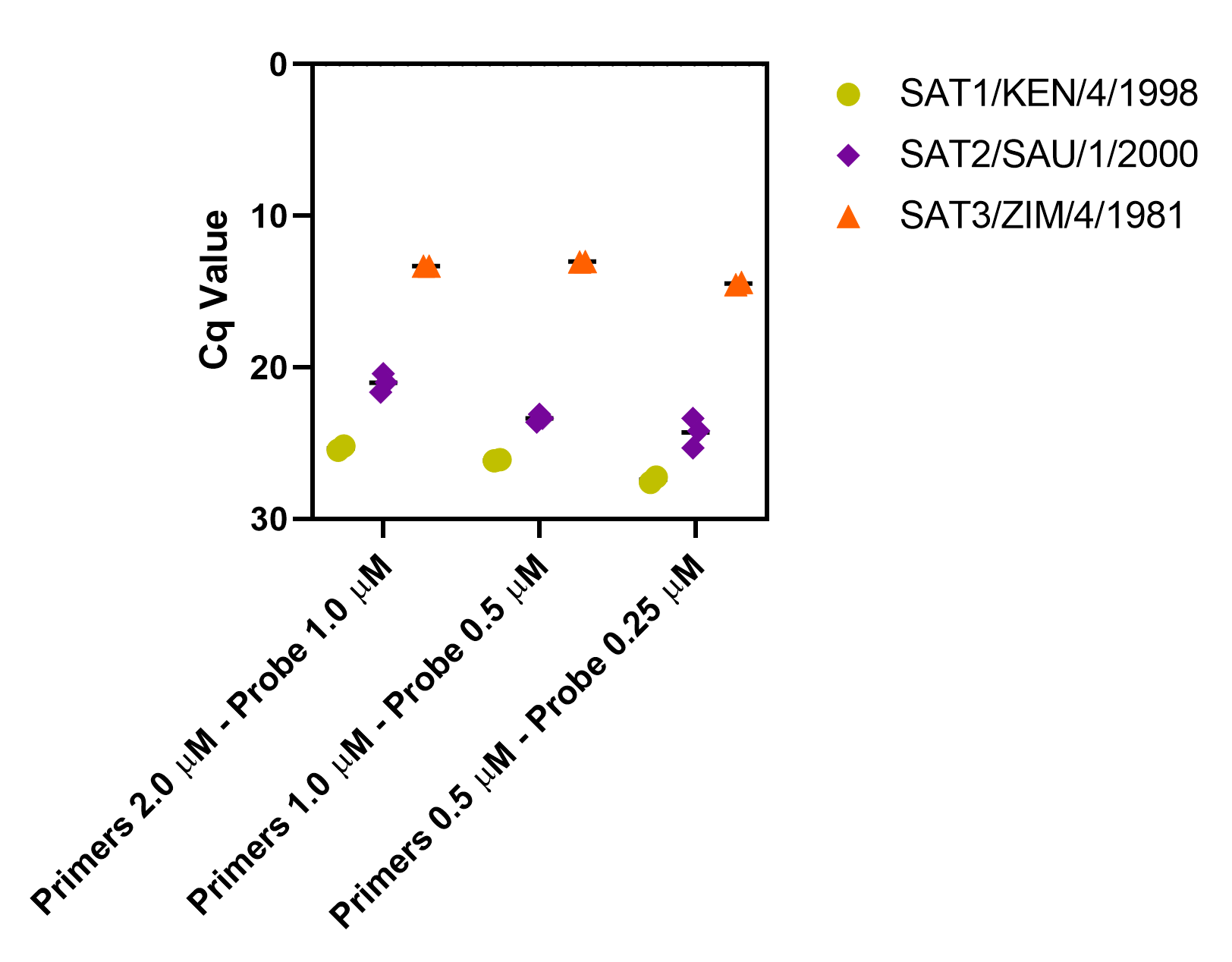

Supplement: Supplementary file 2 [file Image_1.TIF]
